# Oral glycogenic acanthosis: variable clinical presentations in three elderly patients^؆^

**DOI:** 10.1016/j.abd.2026.501429

**Published:** 2026-08-10

**Authors:** Filipe Monteiro, Inês Tribolet-Abreu, Gustavo Almeida-Silva, Pedro de Vasconcelos, Sónia Fernandes, Joana Antunes

**Affiliations:** a Department of Dermatology, Unidade Local de Saúde de Santa Maria, Lisboa, Portugal; b Dermatology University Clinic, Faculdade de Medicina, Universidade de Lisboa, Lisboa, Portugal

Dear Editor,

Glycogenic Acanthosis (GA) is a benign epithelial alteration characterized by intracytoplasmic glycogen accumulation within squamous keratinocytes.^1^ Although classically described in the esophagus, oral involvement is likely underrecognized and may generate clinical concern when morphology is atypical. The authors report three cases of oral GA with distinct clinical presentations, emphasizing histopathologic features and differential diagnosis.

## Case 1

**Fig. 1:**
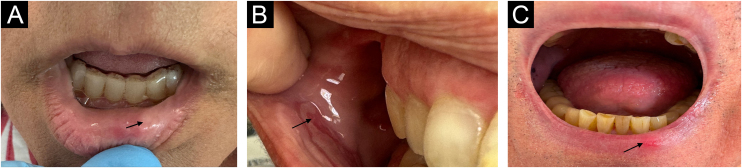
Clinical image: (A) Yellowish plaque on the inner surface of the lower lip (arrow). (B) Erythematous eroded plaque on the buccal mucosa (arrow). (C) Erythematous plaque on the lower lip (arrow).

**Fig. 2:**
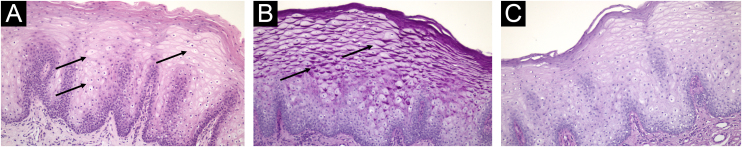
(A) Haematoxylin & eosin, 100 × . Enlarged suprabasal keratinocytes exhibiting abundant pale cytoplasm and small centrally located nuclei (arrows). (B) Periodic acid–Schiff, 100 × . Strong intracytoplasmic positivity (arrows). (C) Periodic Acid-Schiff with Diastase, 100 × . Loss of staining after diastase.

A 58-year-old woman with gastroesophageal reflux disease presented with a long-standing yellowish plaque on the inner surface of the lower lip (Fig. 1A). Histopathological examination showed acanthotic stratified squamous epithelium with elongation of rete ridges and numerous enlarged suprabasal keratinocytes exhibiting abundant pale cytoplasm and small centrally located nuclei. No cytologic atypia, dysplasia, or invasion was identified. No significant alterations were observed in the lamina propria. Periodic Acid-Schiff (PAS) staining demonstrated strong intracytoplasmic positivity that was abolished after diastase digestion (PAS-D), confirming glycogen (Fig. 2).

## Case 2

**Fig. 3:**
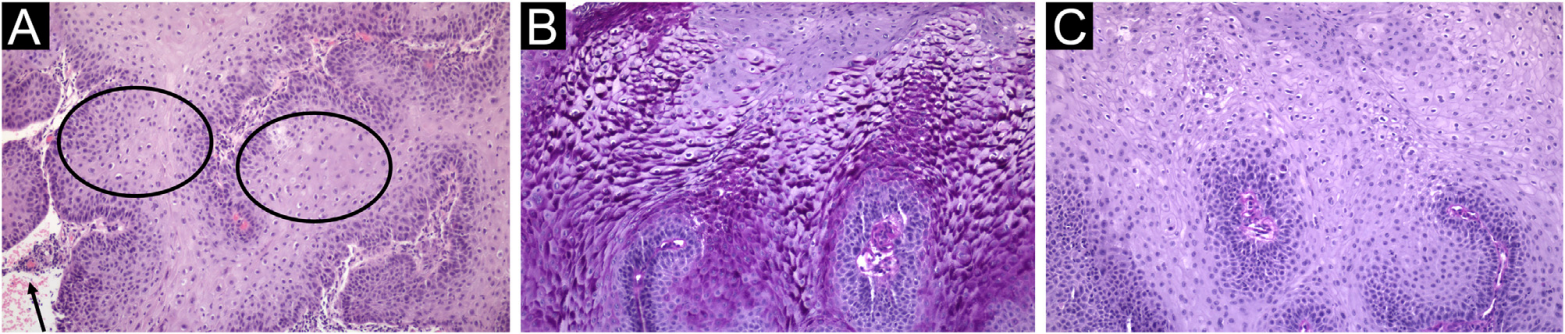
(A) Haematoxylin & eosin, 100 × . Clusters of glycogen-rich clear keratinocytes (circle) and mild perivascular inflammatory infiltrate in the lamina propria (arrow). (B) Periodic acid-Schiff, 100 × . Strong intracytoplasmic positivity. (C) Periodic Acid-Schiff with Diastase, 100 × . Loss of staining after diastase.

A 68-year-old woman with hypertension and dyslipidemia was evaluated for an eroded plaque on the buccal mucosa (Fig. 1B). Because erosion in this location may suggest trauma, inflammatory disease, or early neoplasia, a biopsy was performed. Histology revealed epithelial acanthosis with clusters of glycogen-rich clear keratinocytes identical to the previous case, without dysplasia, and a mild perivascular inflammatory infiltrate in the lamina propria. PAS positivity with loss of staining after diastase confirmed glycogen accumulation (Fig. 3). The surface erosion was interpreted as secondary traumatic change.

## Case 3

**Fig. 4:**
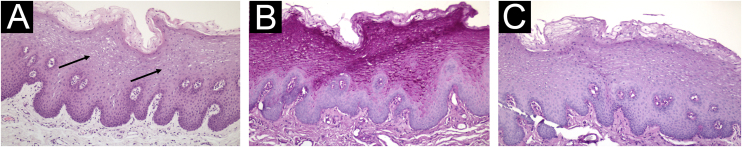
(A) Haematoxylin & eosin, 100 × . Prominent clear keratinocytes lacking nuclear atypia or architectural disturbance (arrows). (B) Periodic acid-Schiff, 100 × . Strong intracytoplasmic positivity. (C) Periodic Acid-Schiff with Diastase, 100 × . Loss of staining after diastase.

A 74-year-old man with hypertension and prior actinic keratosis presented with a chronic erythematous plaque on the lower lip, raising suspicion for actinic cheilitis or squamous cell carcinoma in situ (Fig. 1C). Microscopy examination showed acanthotic squamous epithelium with prominent clear keratinocytes lacking nuclear atypia or architectural disturbance. No basal atypia, disordered maturation, or invasion was observed. The underlying lamina propria was unremarkable. PAS was positive and PAS-D negative (Fig. 4). The erythematous appearance was attributed to vascular prominence and mucosal thinning rather than epithelial dysplasia.

In all cases, enlarged keratinocytes with abundant glycogen-rich cytoplasm were predominantly located in the suprabasal layers, with preserved nuclear morphology. PAS positivity with diastase sensitivity is critical to confirm glycogen and to exclude other causes of clear cell change.

The histopathologic differential diagnoses include: clear cell squamous cell carcinoma, which shows cytologic atypia and invasion; viral lesions with koilocytosis; candidiasis, in which fungal hyphae remain PAS positive after diastase; pagetoid intraepithelial spread of malignancy with mucin positivity; and xanthomatous processes characterized by foamy macrophages in the lamina propria.^2^, ^3^ In addition, inflammatory and potentially premalignant mucosal disorders must be considered clinically. Oral lichen planus may present with white plaques or erosions, but typically demonstrates interface dermatitis with basal vacuolar alteration and band-like lymphocytic infiltrate, absent in these cases.^4^ Leukoplakia, a clinical term for white plaques of uncertain risk, may show hyperkeratosis with or without epithelial dysplasia, but lacks the uniform suprabasal glycogen accumulation characteristic of GA.^5^ Recognition of these distinctions is essential to avoid overdiagnosis.

Clinically, oral GA most commonly presents as plaque-like, slightly elevated white or yellowish lesions, but papular, nodular, and verrucous forms have also been described.^6^ Multifocal or syndromic presentations have been reported, particularly in association with Cowden syndrome, although the strength and consistency of this association remain uncertain.^7^, ^8^ While GA is frequently observed in older individuals and has been regarded as an age-related epithelial change, it can also occur in younger patients, including pediatric cases, suggesting that aging alone does not fully explain its pathogenesis.^9^ Likewise, the relationship between GA and gastroesophageal reflux disease, well documented in esophageal lesions, remains unclear for oral manifestations. Current evidence does not establish GA as premalignant.^3^

These cases illustrate that oral glycogenic acanthosis may present as yellowish papules, eroded lesions, or erythematous plaques mimicking inflammatory or neoplastic conditions. Histopathologic evaluation with PAS and PAS-D staining is decisive for diagnosis. Awareness of its variable clinical spectrum and broad differential diagnosis is important to prevent misinterpretation and unnecessary interventions.

## ORCID ID

Inês Tribolet-Abreu: 0009-0008-4497-9763

Gustavo Almeida-Silva: 0009-0000-0669-5366

Pedro de Vasconcelos: 0000-0002-7480-1228

Sónia Fernandes: 0000-0002-6936-2048

Joana Antunes: 0000-0003-0221-7193

## Financial support

None declared.

## Authors’ contributions

Filipe Monteiro: Data collection, analysis, and interpretation; preparation and writing of the manuscript; approval of the final version of the manuscript.

Inês Tribolet-Abreu: Preparation and writing of the manuscript and approval of the final version of the manuscript.

Gustavo Almeida-Silva: Preparation and writing of the manuscript and approval of the final version of the manuscript.

Pedro de Vasconcelos: Data collection, analysis, interpretation, and approval of the final version of the manuscript.

Sónia Fernandes: Critical literature review and approval of the final version of the manuscript.

Joana Antunes: Critical literature review and approval of the final version of the manuscript.

## Research data availability

Does not apply.

## Conflicts of interest

None declared.
